# Supplementary material for: Mechanical Confinement and DDR1 Signaling Synergize to Regulate Collagen‐Induced Apoptosis in Rhabdomyosarcoma Cells
Source: Adv Sci (Weinh). 2022 Aug 11;9(28):2202552. doi: 10.1002/advs.202202552 (PMC9534977; doi:10.1002/advs.202202552)
Supplement: Supplementary file 1 — Supporting Information [file ADVS-9-2202552-s001.pdf]

ADVANCED SCIENCE
Open Access

Supporting Information

for *Adv. Sci.*, DOI 10.1002/advs.202202552

Mechanical Confinement and DDR1 Signaling Synergize to Regulate Collagen-Induced Apoptosis in Rhabdomyosarcoma Cells

Jordi Gonzalez-Molina, Katharina Miria Kirchhof, Bhavik Rathod, Lidia Moyano-Galceran, Maria Calvo-Noriega, Georgia Kokaraki, Astrid Bjørkøy, Monika Ehnman, Joseph W. Carlson and Kaisa Lehti**

Supporting Information

Mechanical confinement and DDR1 signaling synergize to regulate collagen-induced apoptosis in rhabdomyosarcoma cells

Jordi Gonzalez-Molina, Katharina Miria Kirchhof, Bhavik Rathod, Lidia Moyano-Galceran, Maria Calvo-Noriega, Georgia Kokaraki, Astrid Bjørkøy, Monika Ehnman, Joseph W. Carlson, Kaisa Lehti**

**Fibrillar collagens**						
	**TCGA-SARC**		**Osteosarcoma (GSE21257)**		**Rhabdomyosarcoma (ITCC)**	
**Gene**	**P value**	**Prognosis**	**P value**	**Prognosis**	**P value**	**Prognosis**
**COL1A1**	0.62	NA	0.95	NA	0.48	NA
**COL1A2**	0.62	NA	0.91	NA	0.37	NA
**COL2A1**	0.28	NA	0.74	NA	0.36	NA
**COL3A1**	0.21	NA	0.34	NA	0.01	Favorable
**COL5A1**	0.13	NA	0.94	NA	0.11	NA
**COL5A2**	0.84	NA	0.83	NA	0.11	NA
**COL5A3**	0.85	NA	0.31	NA	0.046	Favorable
**COL11A1**	0.044	unfavorable	0.0105	unfavorable	0.33	NA
**COL11A2**	0.023	unfavorable	0.57	NA	0.2	NA
**COL24A1**	0.0016	unfavorable	0.48	NA	0.73	NA
**COL27A1**	0.0037	unfavorable	0.16	NA	0.054	NA
**Collagen biosynthesis and crosslinking**						
	**TCGA-SARC**		**Osteosarcoma (GSE21257)**		**Rhabdomyosarcoma (ITCC)**	
**Gene**	**P value**	**Prognosis**	**P value**	**Prognosis**	**P value**	**Prognosis**
**ADAMTS3**	0.49	NA	0.0052	unfavorable	0.25	NA
**ARG1**	0.72	NA	0.52	NA	0.93	NA
**BMP1**	0.45	NA	0.61	NA	0.024	Favorable
**FMOD**	0.013	Favorable	0.4	NA	0.63	NA
**LOX**	0.034	Unfavorable	0.5	NA	1	NA
**LOXL2**	0.023	Unfavorable	0.71	NA	0.0496	Favorable
**P3H3**	0.15	NA	0.88	NA	0.0297	Favorable
**P3H4**	0.0024	Unfavorable	1	NA	0.0288	Favorable
**PLOD1**	0.0089	Unfavorable	0.3	NA	0.74	NA
**PLOD2**	0.019	Unfavorable	0.16	NA	0.74	NA
**PLOD3**	0.38	NA	0.97	NA	0.36	NA
**RCN3**	0.083	NA	0.31	NA	0.58	NA
**SERPINH1**	0.097	NA	1	NA	0.2	NA
**TRAM2**	0.23	NA	0.17	NA	0.21	NA
NA: not applicable/ unknown						

Table S1. Prognosis value of fibrillar collagen and collagen biosynthesis genes in sarcoma.

Cell line	Histology	Fusion	P53 status	Metastasis	Location	Age (years)
RD	Embryonal	Negative	Mutant	Primary	Pelvis	7
RH30	Alveolar	Positive	Mutant	Metastasis	B. Marrow	16
RH36	Embryonal	Negative	Wild type	Metastasis	L. Node	15
RMS	Alveolar	Positive	NA	Metastasis	Pleural eff.	14
RMSYM	Embryonal	Multiple chromosomal rearrangements	Mutant	Primary	Abdomen	2
RUCH2	Botryoid	Multiple chromosomal rearrangements	NA	Primary	Vagina	1
NA: not applicable/ unknown						

Table S2. Rhabdomyosarcoma cell line details.

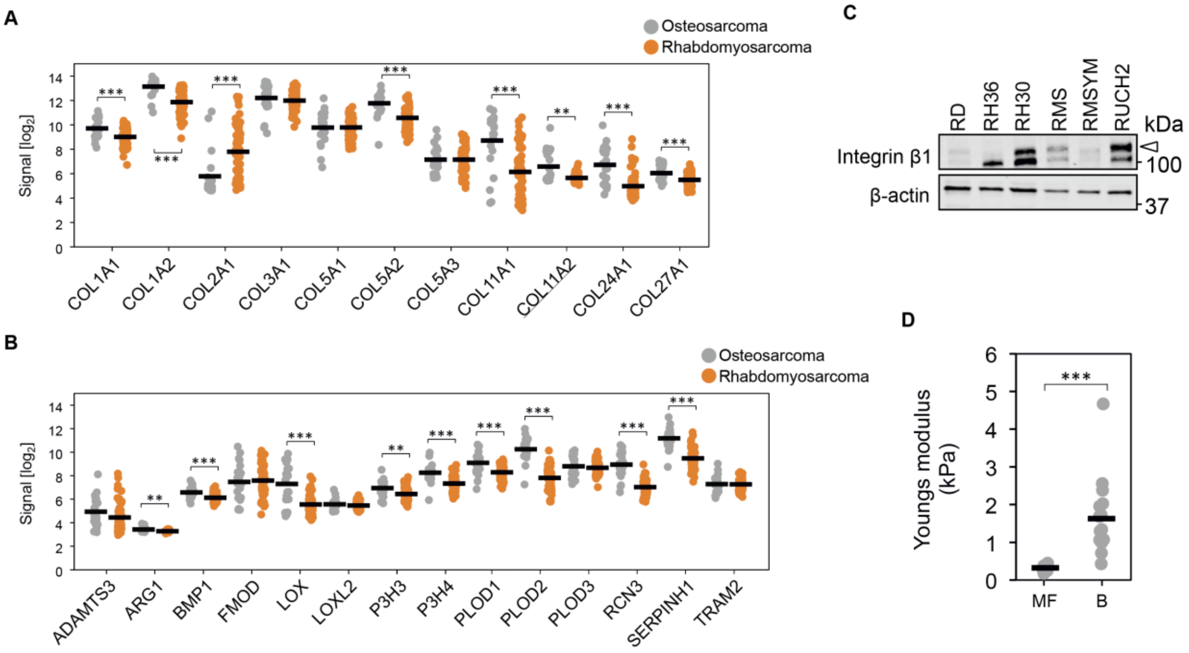

Figure S1. Expression of collagen-related gene and integrin β1 protein in RMS and stiffness of collagen matrices with distinct microarchitecture. **A**, **B**, Gene expression of fibrillar collagens (A) and collagen biosynthesis-related genes (B) in osteosarcoma (OS; n=21; GSE87437) and rhabdomyosarcoma (RMS; n=58; GSE66533) tumors. **C**, Integrin β1 protein expression in RMS cell lines. Arrowhead indicates the band corresponding to mature, fully glycosylated, integrin β1. **D**, Young's moduli of microfibrillar-collagen (MF; n=17 spots) and bundle-collagen (B; n=16 spots). Each data point is shown, bars indicate average Young's moduli. *** $p < 0.001$, ** $p < 0.01$.

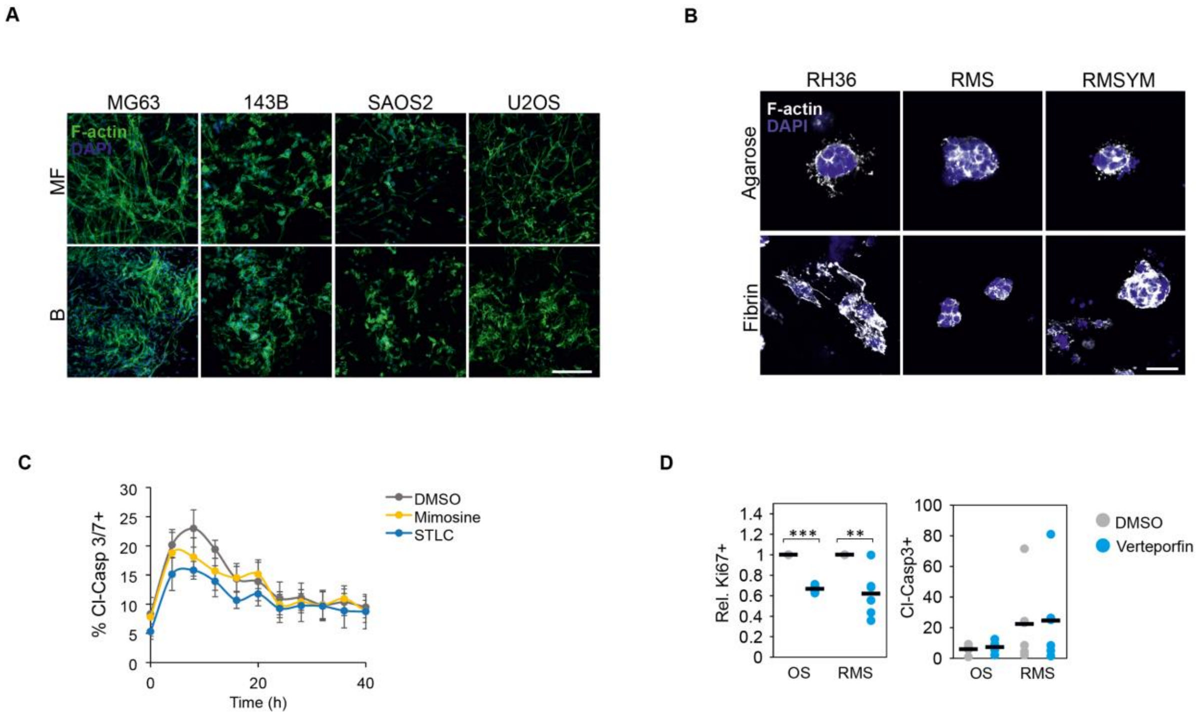

Figure S2. 3D collagen matrices induce apoptosis in RMS cell lines independently of cell cycle and YAP activation. **A**, Representative images of OS cell lines embedded in microfibrillar (MF)- or bundled (B)-collagen for 4 days showing similar cell density in both matrices. Scale bar indicates 50 µm. **B**, Collagen-apoptotic RMS cell lines form spheroids in soft agarose and fibrin matrices after 4 days. Scale bar indicates 10 µm. **C**, Blocking cell cycle progression does not alter apoptosis dynamics induced by 3D MF-collagen on RH36 cells (n=3 biological repeats). **D**, Treatment with the YAP-TEAD inhibitor verteporfin reduces proliferation but does not alter apoptosis in cells embedded in 3D MF-collagen for 24 h. Each data point indicates average (n=3 biological repeats) of a cell line (OS n=4 cell lines, RMS n=6 cell lines). *** $p < 0.001$, ** $p < 0.01$.

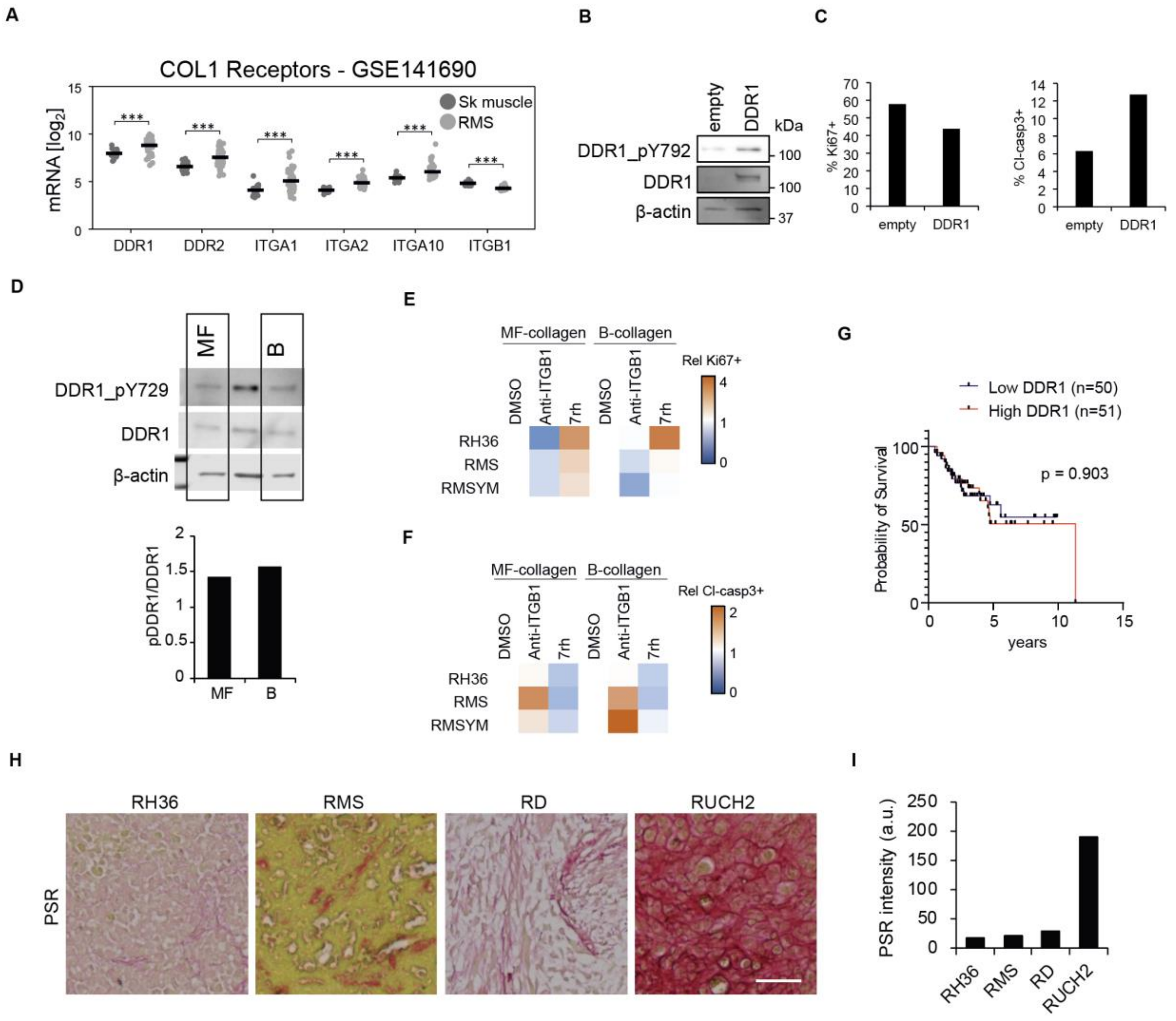

Figure S3. Inhibiting DDR1 reduces and blocking integrin β1 enhances the apoptotic response to 3D collagen in RMS cells. **A**, Comparison of collagen I receptor gene expression between skeletal muscle (Sk muscle; n=9) and rhabdomyosarcoma (RMS) tumors (n=66) showing a general increase except for ITGB1 in tumors tissues. **B**, Western blot analysis showing the efficiency of DDR1 overexpression in RH30 cells. **C**, Effect of DDR1 overexpression on RH30 cell proliferation (Ki67+) and apoptosis (Cleaved-caspase 3+) after 24 h embedding in 3D microfibrillar (MF)-collagen (n=3 technical repeats). **D**, Uncut blots and quantification of phosphorylated DDR1 to DDR1 ratio in RMSYM cells adhered to MF- or bundled (B)-collagen for 24 h (n=2 biological repeats). **E**, **F**, Variation in cell proliferation (E) and apoptosis (F) in collagen-apoptotic RMS cell lines embedded in 3D MF-collagen for 24 h upon the indicated treatment showing opposite effects of DDR1 and integrin β1 inhibition (n=3 biological repeats). **G**, Kaplan-Meier survival curves of RMS tumors expressing low (n=50) or high (n=51) DDR1 (from the ITCC cohort) showing no difference

between these groups. **H**, **I**, Representative images (H) and quantification (I) of collagen content (PSR staining) in rhabdomyosarcoma tumor xenografts of collagen-apoptotic and collagen-proliferative cell lines. Scale bar 50 μm. *** $p < 0.001$.

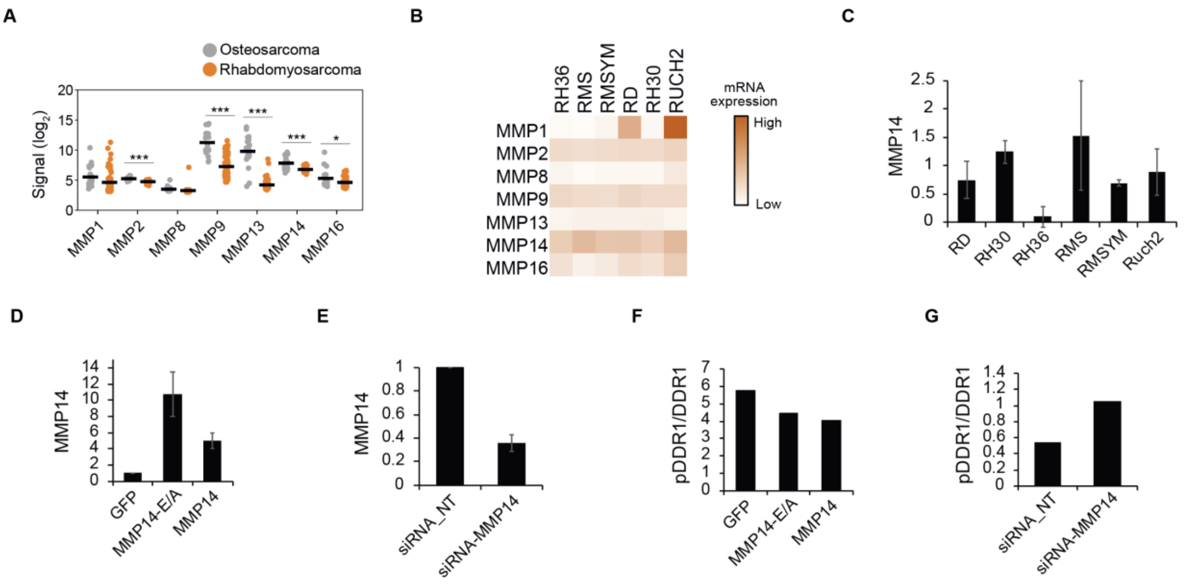

Figure S4. Matrix metalloproteinase gene expression is relatively low in RMS cells. **A**, Comparison of collagen-proteolytic matrix metalloproteinase (MMP) gene expression between OS (n=21; GSE87437) and RMS (n=58; GSE66533) tumors. **B**, Gene expression of collagen-proteolytic MMPs in RMS cell lines. **C**, Quantification of MMP14 protein levels per cell line (n=3 biological repeats). **D**, **E**, Quantification of MM14 levels in RH36 cells transfected with control (GFP), catalytically inactive MMP14 mutant (MMP14-E/A) and wild-type MMP14 (MMP14) (D, n=3 biological repeats) and in RH30 cells after MMP14 knockdown (siRNA_MMP14) or non-targeting control (siRNA_NT) (E, n=3 biological repeats). **F**, **G**, Quantification of phosphorylated DDR1 to DDR1 ratio of cells from (D) (F, n=1) and (E) (G, n=1). Bar graphs represent average (±S.E.M.).

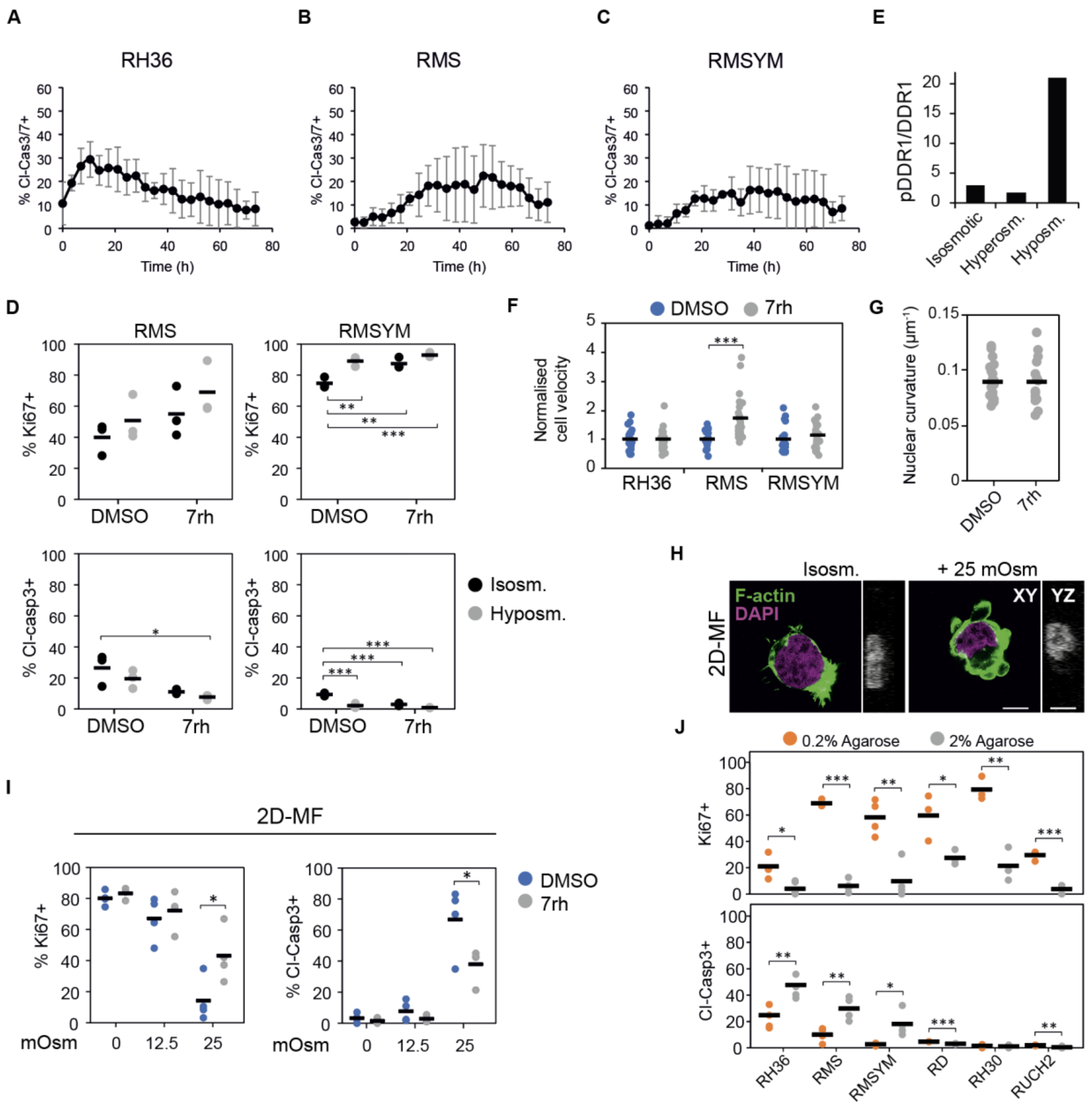

Figure S5. Mechanical confinement synergizes with DDR1 signaling to induce apoptosis in RMS cells. **A-C**, Dynamics of apoptosis induced by MF-collagen in RH36 (A), RMS (B), and RMSYM (C) cells (n=5 technical repeats). **D**, Hyposmotic pressure and DDR1 inhibition synergize to enhance proliferation and reduce apoptosis in RMS and RMSYM cells embedded in 3D microfibrillar (MF)-collagen for 24 h (n=3 biological repeats). **E**, Quantification of phosphorylated DDR1 to DDR1 ratio in RH36 cells adhered to 2D MF-collagen for 6 h (n=2 biological repeats). **F**, Cell velocity normalized to control (DMSO) of cells embedded in 3D MF-collagen during the first 5 h (n≥21 cells). **G**, Nuclear curvature of RH36 cells embedded in 3D MF-collagen for 3.5 h (n≥14 cells). **H**, Representative images of

RH36 cells on 2D MF-collagen gels subjected to distinct osmotic pressures showing membrane blebbing and changes in nuclear morphology in hyperosmotic conditions. Scale bars indicates 10 μm. **I**, Inhibition of DDR1 reduces apoptosis in RMS cells seeded on 2D MF-collagen subjected to hyperosmotic conditions (n=3 biological repeats). **J**, Apoptosis is enhanced in collagen-apoptotic RMS cells lines embedded in high density agarose compared to low density agarose hydrogels after 24 h (n=3). *** $p < 0.001$, ** $p < 0.01$, * $p < 0.05$.

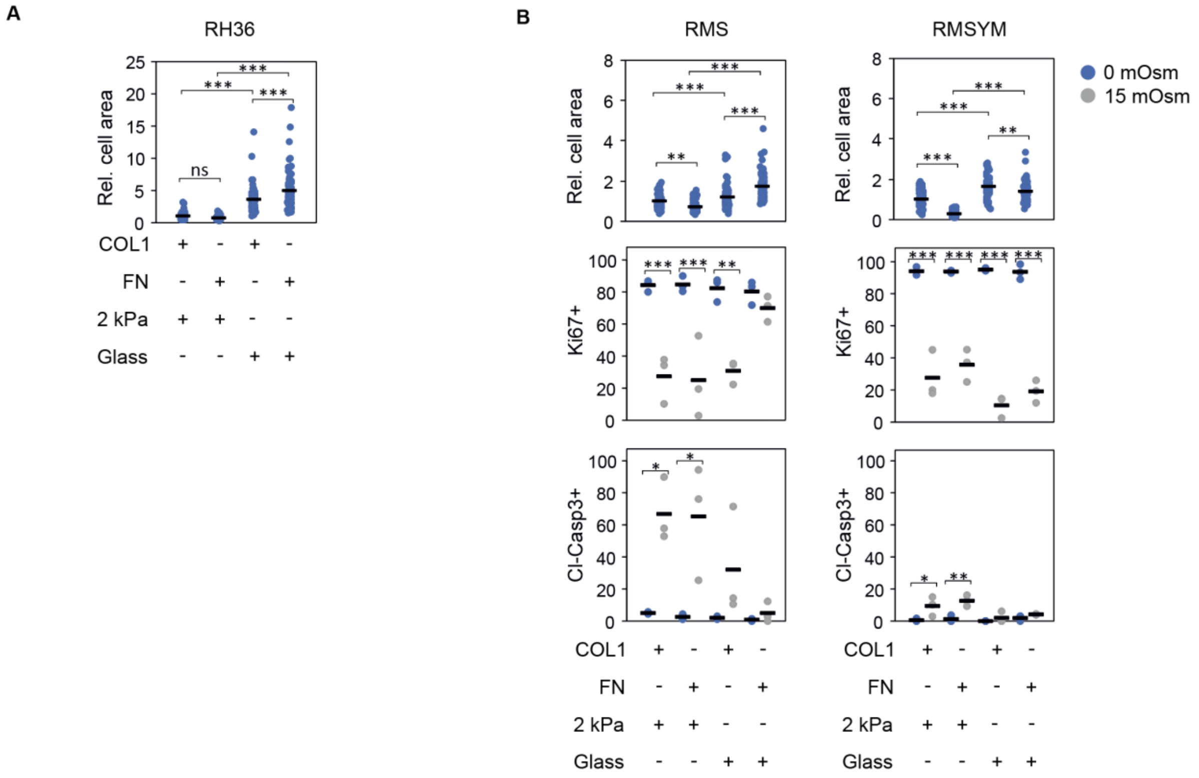

Figure S6. Enhanced cell adhesion reduces hyperosmotic pressure-induced apoptosis. **A**, Effect of adhesion to distinct substrates on RH36 cell spreading (n≥74 cells). **B**, Effect of cell adhesion to distinct substrates on RMS and RMSYM cell spreading (top panels; n≥75 cells), proliferation (middle panels; n=3 biological repeats), and apoptosis (bottom panels; n=3 biological repeats). *** $p < 0.001$, ** $p < 0.01$, * $p < 0.05$.

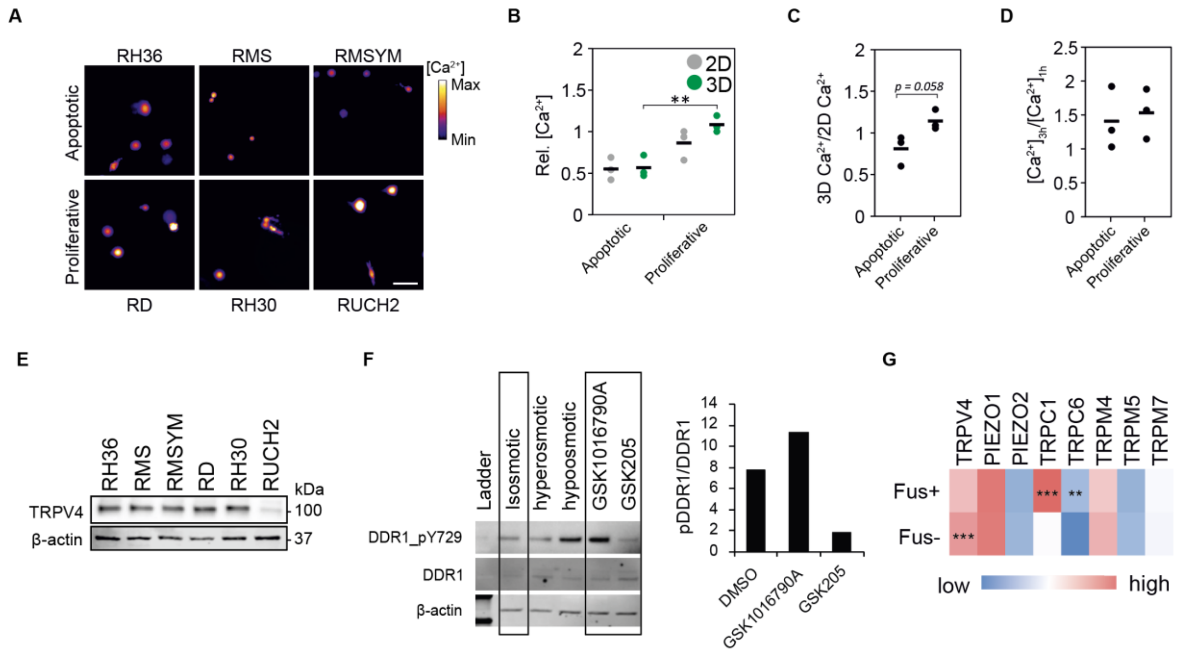

Figure S7. Intracellular calcium content and TRPV4 expression comparison in RMS cell lines. **A**, Representative images of relative calcium content in RMS cell lines embedded in 3D microfibrillar (MF)-collagen for 3 h. Scale bar indicates 20 µm. **B**-**D**, Comparison of the relative calcium content (B), or the ratio of calcium content in cells on 2D and in 3D MF-collagen (C) after 3 h or the difference in calcium content between 1 h and 3 h after embedding (D) in collagen-apoptotic and collagen-proliferative cell lines. Each data point indicates the average of one cell line and bars show the average of each group. **E**, Protein expression of TRPV4 in the distinct RMS cell lines. **F**, Uncut blots and quantification of phosphorylated DDR1 to DDR1 ratio in RH36 cells adhered to MF-collagen for 6 h under the indicated treatments (n=1). **G**, Gene expression of mechanosensitive channels in fusion-positive (Fus+) and fusion-negative (Fus-) RMS tumors (ITCC cohort). *** $p < 0.001$, ** $p < 0.01$.
